# Virtual Care and Mental Health: Dismantling Silos to Strengthen Care Delivery

**DOI:** 10.1089/tmr.2023.0016

**Published:** 2023-07-11

**Authors:** John Scott, Peter Yellowlees, Daniel F. Becker, Christopher Chen

**Affiliations:** ^1^ Department of Medicine, Allergy and Infectious Diseases, University of Washington, Seattle, Washington, USA.; ^2^ Department of Psychiatry, University of California Davis, Sacramento, California, USA.; ^3^ Department of Psychiatry, University of California San Francisco, San Francisco, California, USA.; ^4^ Department of Psychiatry, Washington State Health Care Authority, Olympia, Washington, USA.

**Keywords:** behavioral health, tele psychiatry, virtual care

## Abstract

**Background:**

During the COVID-19 pandemic, many Americans experienced new or worsened mental health conditions. Concurrently, much care switched from in-person to virtual care, highlighting the value of virtual care but also some of the underlying challenges.

**Methods:**

This paper explores one such challenge, the separation of mental health care from physical health care, and a potential solution, collaborative care. It is a team-based approach linking psychiatrists to primary care providers that can help break down the silos of care created through reimbursement models.

**Results:**

In this context of collaborative care, high quality virtual care further bridges the divide between physical and mental health care. Asynchronous virtual care for mental and behavioral health is an innovation that can create efficiencies while still supporting collaborative care.

**Discussion:**

The barriers and weaknesses of using virtual care exclusively for mental and behavioral health are discussed, as well as examples of policy changes which can improve mental health care through collaborative virtual care.

The COVID-19 pandemic was an incredibly stressful and destabilizing event for much of society, resulting in social isolation and higher rates of mental disorders. A Kaiser Family Foundation survey found that >40% of American adults reported feelings of anxiety or depression in 2020, compared with 11% prepandemic.^1^ Fortunately, health care providers and systems were able to pivot rapidly to the use of virtual care in the care of patients with mental and behavioral disorders. Although virtual care was a fairly common care delivery modality before the pandemic, it skyrocketed during the pandemic.

In a national study of >60 private insurers, there was a 2816% increase in virtual care consultations by end of 2020; almost half of these virtual visits were for mental health.^2^ During the early phases of the pandemic, >90% of outpatient mental disorder care at our three academic institutions was provided virtually and this rate has been maintained to this day (internal information, unpublished).

Virtual care for mental and behavioral disorders is not a panacea for patients or the American health system. The pandemic highlighted some of the well-known deficiencies in the mental health system, in particular the fact that much of the care occurs in a silo, disconnected from the care of other health conditions.^3^

In this article, we explore the genesis of mental health care as a carveout, the use of collaborative care to bridge this gap, the reinforcement of collaborative care through virtual care, the limitations of virtual care for mental and behavioral disorders, and then with examples of policy changes that have strengthened virtual care delivery and collaborative care overall. For the purposes of this discussion, we are focused on the use of virtual care for the diagnosis and treatment of mental and behavioral health conditions in adults by psychiatrists.

## Silos of Health Care: Mental Health Versus Physical Health

When managed care became a common way of financing medical care in the 1980s, one of the tools for managing behavioral health care was to “carve” it out.^4^ In other words, the financial management of psychiatric care was placed in a different company, perhaps a subsidiary of the larger insurance company or different agency that was then governed under separate rules such as limiting patients to a set number of counseling sessions per year, or lifetime often with lower annual caps.

The pendulum swung back to “carve in” some behavioral health benefits in the 1990s but then other mental health benefits were “carved out” again in the 2000s through laws such as California's *AB 88* that took certain diagnoses and moved them into a category that required coverage at levels similar to nonpsychiatric conditions.^5^

The consequence, unfortunately, is that because many payor systems are still separated for psychiatric versus nonpsychiatric conditions, there are silos of care, making it difficult for patients to access help and for providers to be reimbursed appropriately. As a result of this insurance disintegration, there has been less focus on screening, early intervention, and prevention in psychiatry than we have seen in other areas of health care. These approaches—often implemented in the primary care setting—have been extremely helpful during the past few decades with respect to reducing the burden of illness in other areas of medicine, but have largely been unavailable for patients with psychiatric disorders.

## Collaborative Care Bridges the Gap

The collaborative care model is an innovation that leverages psychiatrists and mental health professionals time and expertise through team-based care. In collaborative care, a team of mental health professionals support primary care physicians and advanced practice providers in primary care. The five principles of collaborative care are (1) patient-centered team care, (2) population-based care, (3) measurement-based treatment to target, (4) evidence-based care, and (5) accountability.^6^

Sometimes also known as “stepped care,” the model encourages and supports primary care providers (PCPs) to manage mild conditions, with support from experts, with only the most complex patients being sent to psychiatric providers for treatment. Once stabilized, these patients are returned to their PCPs, allowing access for the next patient.

Collaborative care originally started out in person, but during the pandemic much of the care and coordination has moved to virtual. The benefits of the model are earlier behavioral care, greater efficiency, and providers working at the top of their scope of practice. Of note, collaborative care can be delivered in real time, asynchronously, or through remote patient monitoring. Medicare has covered collaborative care since 2016. Washington state was the first Medicaid program to formalize sustainable reimbursement for collaborative care in 2018, and at least 17 state Medicaid programs currently reimburse for this model of care.^7^

Psychiatry was one of the early specialties to adopt the use of virtual care. Among the benefits documented in studies are reduced costs to patients and providers, high patient satisfaction, and improved access to underserved and remote communities.^8,9^ The clinical interview also does not require physically touching the patient that makes adoption of virtual interviews more natural and without a diminution in clinical quality. Furthermore, psychiatric providers are now able to see the patients in their lived environment at home or in community settings and thereby better understand their support systems.

During the public health emergency, home finally became an accepted site of service for Medicare patients, with most private insurances following suit, and reimbursement and licensing regulations were relaxed, making virtual care for mental health and substance use disorders much more accessible. Excellent guidelines for clinicians have been available from both the American Telemedicine Association and the American Psychiatric Association for many years.^10^

Patients with psychiatric disorders have faced considerable stigma over many years, creating barriers to care although this has lessened in recent times. Combined with the availability of good pharmacological and nonpharmacological interventions such as cognitive behavioral therapy, the demand for mental health care has skyrocketed, outpacing the supply of trained psychiatric specialists. It is estimated that in the coming years, there will be an expected shortfall of between 14,000 and 31,000 psychiatrists, psychologists, and social workers.^11^

In addition, that workforce tends to be concentrated in urban areas. For example, in Washington State, 17 of 38 (45%) of the counties have no psychiatrist.^12^ Part of the issue is attributable to population density: in the more rural areas, it is hard to have the right provider there at the right time because the numbers are just too small. Virtual care helps overcome these barriers by allowing psychiatrists to see patients in more rural and remote areas of their state. In addition, patients may encounter less stigma in seeking care because they do not physically have to go to a psychiatrist's office or mental health care facility.

One well-described use case of the collaborative care model is for secondary or complex care of patients with chronic medical conditions, such as diabetes mellitus or heart failure, who have associated mental health issues that can adversely impact their physical health. For this reason, health systems increasingly have psychiatric care integrated into the care of patients with chronic medical conditions.

Another example of collaborative care through virtual modalities is in the treatment of patients with addictions and behavioral disorders. When treating patients with substance use disorders, specialists use considerable interdisciplinary collaboration, and it is critical to get the right team together in the right place and at the right time with the patient. In person, this can be challenging, but when done virtually it becomes much easier and with less stigma. Some patients with addictions are reluctant to walk into a clinic setting that is associated with addiction treatment.

Equally, there are people who are hesitant to do a video visit from their home because there are others in the home who are unaware of the addiction treatment. Being mindful of the latter situation is important for psychiatric providers when setting up virtual visits, and finding neutral sites that are private and have technological support may require some additional time.

## Asynchronous Virtual Care: An Efficient Method to Support Collaborative Care

Asynchronous telemedicine for mental health conditions presents a major opportunity for improved and more efficient care while still supporting the collaborative care approach. One model is the eConsult system, in which a provider sends a request to a psychiatric specialist through the electronic medical record.^13^ Much of the relevant data, like the patient health questionnaire (PHQ-9), are pulled from the electronic medical record (EMR) automatically.

Specialists then review the question, relevant data, and write back with an impression and recommendation, which the requesting provider may or may not choose to implement. PCPs are able to work at the top of their license and provide mental health care for less complicated patients through this supportive model. In the United States, Medicare started paying for this service in 2018 and many private insurance and state Medicaid programs now compensate for this as well.

**FIG. 1.**
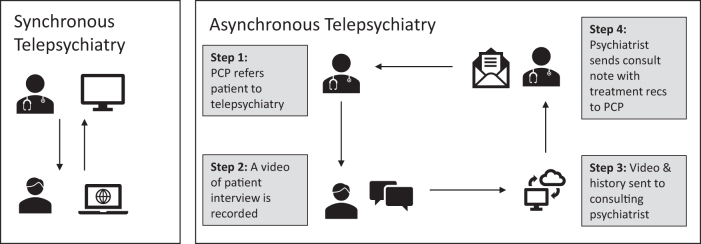
Differences in workflow for synchronous vs. asynchronous telepsychiatric care (adapted from Yellowlees^14^).

A more detailed approach to asynchronous care has been described by Yellowlees et al.^14^ and Chan et al.^15^ In this process (Fig. 1), someone other than the psychiatrist interviews the patient, records that interview, and sends the video to a psychiatrist who then writes a consultation note with treatment recommendations. Two studies have shown this approach to be just as effective as traditional synchronous telepsychiatry^16,17^ and to achieve similar PCP adherence to recommendations (∼60%).^18^

The process requires training the interviewer and needs to be done in patient's native language. Fortunately, this type of asynchronous care has been shown to be effective for Spanish-speaking patients, but one should always use professional medical interpreter services for interpretation into and out of English since commonly used translation apps are not adequate for psychiatric and medical care.^19^

## Barriers and Limitations of Virtual Care

Although virtual care in general works well for psychiatric care, it is not the right modality of care for every patient or every interaction. For example, patients who are new to a clinician, very young, presenting with an acute issue, or without reliable access to high-speed internet may not be ideal candidates for a telemedicine visit. In addition, some psychiatric professionals feel like it is more difficult to establish a therapeutic relationship virtually as compared with in person,^20^ although conversely there is a substantial group of patients who, because of stigma, will not personally visit a psychiatrist, but are prepared to see them virtually.

Regulatory hurdles present another significant challenge for the use of telemedicine for the care of patients with mental and behavioral disorders. The Ryan Haight Act was created in 2008 in an attempt to shut down overseas “pill mills,” especially for controlled substances such as opioids and benzodiazepines.^21^ It requires health care providers to conduct at least one medical evaluation in the physical presence of the patient before prescribing controlled substances.

Although there are some exceptions for this requirement, the policy does not account for current workflows and realities of telemedicine delivery for patients with mental health and substance use disorders. The drug enforcement agency (DEA) has been tasked by congress to develop a special registration program for telehealth providers but has not yet created it. With the ending of the federal public health emergency (PHE) in May 2023, providers will face the uncertainty of Ryan Haight Act requirements again in place, and at the time of writing the DEA has just published draft recommendations that require an initial in-person evaluation before prescribing controlled substances, an overtly retrograde step from a clinician's perspective.

This means that for many patients, it may require a lengthy and expensive trip to the clinician's office, whereas for providers, many of their clinical spaces have been either reconfigured for other uses or they have moved to other parts of their state to reduce living expenses, conducting most of their visits virtually.

Finally, from a policy and payment perspective, there is a lack of data infrastructure to ascertain effectiveness of virtual care for mental and behavioral disorders on a population level. This makes it difficult for payors to judge whether a large-scale change, such as certain use cases of telemedicine, is safe and clinically effective.

## Policy Interventions

Policy level interventions are necessary to maximize the benefit of telemedicine for psychiatric care. For example, “parity laws” that treat the provision of care by telemedicine on par with in-person care are vitally important. Washington State passed a law in 2020 (SB 5385) that permanently required that Medicaid and commercial plans reimburse providers for telemedicine visits at the same rate as in-person care.^22^ This law was critical for the rapid expansion of telemedicine during the COVID-19 pandemic.

There is much opportunity for policy changes at the state level. For example, the Washington State Health Care Authority fully integrated physical and behavioral health under the Medicaid program in 2016.^23^ This means that services are coordinated through a single health plan including physical health, mental health, and substance use treatment. Washington Medicaid telehealth policy has additionally been flexible and adaptable, authorizing reimbursement for eConsults, portal visits, audio only, and remote patient monitoring in the past 5 years.

In addition, the health care authority has helped both providers and patients with technology needs. It directly provided >2000 free Zoom licenses to providers in Washington State, with the vast majority of them, >70%, being utilized by behavioral health providers that proved to be a critical lifeline to be able to continue delivering services during the pandemic.^24^ For patients, it issued 6000 cell phones and >800 laptops to help them conduct telemedicine visits with their providers.

Partnerships have been critical for the success of telemedicine programs in Washington State. The University of Washington Behavioral Health Institute provided technical assistance directly to behavioral health providers who needed to adapt their different models of care and clinical workflows and also partnered with managed care organizations and their telehealth vendors to facilitate the rapid shift to telehealth. Through the funding from the state, the University of Washington (UW) and Seattle Children's Hospital stood up a free consult line, which operates 24/7 and provides primary care providers with a direct line to consulting psychiatrists as well as the partnership access line that is for children and adolescents.

Finally, the health care authority works in partnership with the state department of social and human services research data analytics group to continue looking at what quality measures can be developed and in a more robust way in behavioral health.

## Summary

The COVID-19 pandemic changed the health care system in the United States in both good and bad ways. One of the positive outcomes was the rise of virtual care, especially for the care of mental health services. Importantly, virtual care supports the collaborative care model for the care of patients with mental and behavioral disorders. Many of the changes in policies and regulations in Washington State are presented as a model for other states. These include support for technological needs by patients and providers, and most importantly, a reimbursement model that integrates mental and physical health. We found that we served our patients best when we thought of virtual care not really as a model of care but as a tool for providing better care.

This frame of reference challenges our assumptions around payment models. Simply put, virtual care alone is not going to solve the challenge of poor access to mental and behavioral health care. It must be coupled with policies that break down the silos of care between mental and physical health and that allows providers to have the flexibility that they need to communicate with clients while complying with preset parameters that ensure quality of services. Lastly, the COVID-19 pandemic showed us that we cannot anticipate the innovations that are happening at the clinical level, such as asynchronous telepsychiatry, and having a flexible payment policy enables such innovations to happen.

## Abbreviations Used

DEA: drug enforcement agency
PCPs: primary care providers
